# Characterization of Polysaccharides Sequentially Extracted from *Allium roseum* Leaves and Their Hepatoprotective Effects against Cadmium Induced Toxicity in Mouse Liver

**DOI:** 10.3390/antiox11101866

**Published:** 2022-09-21

**Authors:** Nesrine Teka, Fahad M. Alminderej, Ghada Souid, Yassine El-Ghoul, Didier Le Cerf, Hatem Majdoub

**Affiliations:** 1 Laboratory of Interfaces and Advanced Materials, Faculty of Sciences of Monastir, University of Monastir, Monastir 5000, Tunisia; 2 Department of Chemistry, College of Science, Qassim University, Buraidah 51452, Saudi Arabia; 3 Research Unit: Mycotoxins, Phycotoxins and Associated Pathologies, Faculty of Pharmacy, University of Monastir, Monastir 5000, Tunisia; 4 Textile Engineering Laboratory, University of Monastir, Monastir 5019, Tunisia; 5 Normandie University, UNIROUEN, INSA Rouen, CNRS, PBS, UMR 6270 & FR 3038, 76000 Rouen, France

**Keywords:** *Allium roseum*, polysaccharides, physicochemical characterization, antioxidant properties, hepatoprotective activity

## Abstract

*Allium roseum* is one of the medicinal plants of the Liliaceae family, widely used in the food industry and traditional medicine. It is known for its various biological properties, such as its antioxidant, antiviral, antidiabetic, and anti-inflammatory activities. The present work aims to extract the polysaccharides from *Allium roseum* leaves and evaluate their antioxidant activities and hepatoprotective effects in vivo. Three polysaccharides from the leaves of *Allium roseum* were sequentially extracted in three media: water, chelating, and basic, respectively. They were characterized by size exclusion chromatography, gas chromatography mass spectrometry, FTIR-ATR, and NMR spectroscopy (1D and 2D). The different polysaccharides principally consist of glucose, galactose, mannose, rhamnose, xylose, and galacturonic acid. The antioxidant activity and hepatoprotective effect of the extracts against Cd-caused oxidative stress in liver mouse were tested. Cd treatment, during 24 h, enhanced significantly lipid peroxidation by a high production of malondyaldehyd (MDA) and superoxide dismutase (SOD) activity. In contrast, catalase activity (CAT) was decreased after the same period of exposure to the metal. The polysaccharides pre-treatment improved the antioxidant defense system to a great degree, mainly explained by the modulating levels of oxydative stress biomarkers (MDA, SOD, and CAT). This research clearly shows that *Allium roseum* polysaccharides, especially those extracted in aqueous medium, can be used as natural antioxidants with hepatoprotective properties.

## 1. Introduction

Reactive oxygen species (ROS) are the most important of the free radicals in biological systems. Still, a build-up of ROS in cells may destroy DNA, RNA, and proteins. It may cause cell death, which would lead to the development of chronic disease conditions, such as cardiovascular, Alzheimer’s, neurological diseases, and the aging process [1].

Cadmium (Cd) is considered one of the widespread toxic metals in aquatic and terrestrial environments [2,3]. Cadmium mainly comes from the Earth’s crust through volcanic eruption and industrial pollution, which causes water and as soil pollution [4]. Cadmium accumulation through food chain and Cd exposure causes a high risk for human health [5,6] and leads to a large variety of diseases, such as hepatic damages [7], as reported by the World Health Organization (WHO) [8]. Indeed, the liver is the organ most sensitive to cadmium toxicity, since it is responsible for the detoxification process in various metabolic pathways [9].

Moreover, Cd is considered one of the reasons for ROS production, such as superoxide anion and hydroxyl radicals. Several studies affirmed that acute Cd exposition leads to the oxidative damages caused by the antioxidant defense system’s imbalance between ROS generation and neutralization. Therefore, Cd exposure provoked several changes in antioxidant enzyme activities, such as catalase (CAT), superoxide dismutase (SOD), and glutathione (GSH), which are used as a biomarkers of metal oxidation [10]. It should be noted that the membrane lipid will undergo peroxidation if the ROS are not neutralized, and an overproduction of malondialdehyde (MDA) will occur.

Over the past few years, the use of medicinal plants as beneficial remedies has been widely increased in the world [11]. They are considered a source of natural antioxidant substances, such as polyphenols and polysaccharides [12]. Thus, reports demonstrated that natural polysaccharides have received increasing attention, due to their diverse biological activities, including antioxidant, antibacterial, antihyperlipidemic, antidiabetic, antiproliferative, and antitumor activities [13,14,15,16]. They are considered more effective and safe, compared to some synthetic antioxidants [17]. Indeed, the polysaccharides extracted from Periploca Angustifolia evinced several protective effects against Cd-induced liver damage in rats [18]. Besides, these inhibit oxidative damage in renal tissues [19]. *Alliums* is one of the very well-known and widely used genera in the world. It is widespread mostly in central Asia, Europe, North America, and Africa [20]. It has been used since antiquity as a condiment and preservative in the food industry [21] and traditional medicine, such as as antiviral, antidiabetic, anti-inflammatory, anticancerous, and antifungal agents, thanks to its various strong biological properties [22]. The *Allium* genus is one of the major sources of natural antioxidant products, due to its polyphenolic and polysaccharides compounds [23]. *Allium roseum* is one of the important wild medicinal plants. It is widely spread throughout Tunisia, mainly found in light and sandy soils. Some studies have demonstrated the essential oil chemical composition of this species [24]. In fact, it has been claimed by many reports that the active substances from the leaves and bulbs of *Allium roseum* contain important biological activities [25]. However, little information is available concerning the antioxidant activities and antiradical potentials of Tunisian Rosy garlic. Recently, Souid et al. [26] studied the anticancer activity of Rosy garlic extracts. However, no studies have reported on polysaccharides extracted from *Allium roseum*. Hence, the purpose of this work has been one to probe the potential protective effect against cadmium-induced damages in mouse liver of the polysaccharides isolated from leaves of *Allium roseum* in different media (water, chelating, and basic) using quantification of antioxidant enzyme activities (CAT and SOD) and the evaluation of the lipid peroxidation (MDA production). Furthermore, the different polysaccharides were characterized by several techniques: GC/MS, FT-IR, size exclusion chromatography, and 1D and 2D NMR.

## 2. Materials and Methods

### 2.1. Material

Leaves of *Allium roseum* were collected from the region of Monastir (Tunisian Sahel; coordinates: lat 35°73′ N; long 10°76′ E) during the flowing period (Marsh), 2017; next, washed with distilled water and ground by a mixer blender and freeze-dried, with the dry powder stored until used. The botany department (Faculty of Pharmacy of Monastir) identified the plant. Trifluoroacetic acid (TFA) and monosaccharide standards (D-Gal, L-Fuc, L-Rha, D-Man, D-Xyl, and D-Glc) were purchased from Sigma–Aldrich (St. Louis, MO, USA).

### 2.2. Preparation of Alcohol Insoluble Solids (AIS)

A total of 27 g of dry powder was treated with 700 mL of boiling ethanol (96%). The mixture was kept boiling for 30 min, and then kept at 24 °C for 24 h. Finally, the obtained residue was dried at a temperature of 40 °C for 48 h.

### 2.3. Extraction and Purification of Polysaccharides from Allium roseum Leaves

**Figure 1:**
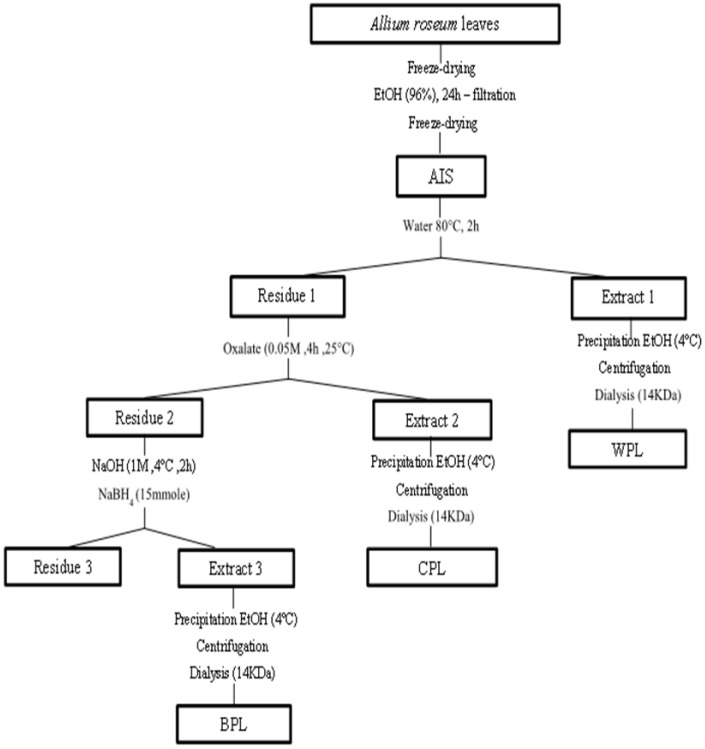
The sequential extraction of polysaccharides from *Allium roseum* leaves.

The polysaccharides were extracted from *Allium roseum* leaves, as proposed by Chaouch et al. and Kratchanova et al., with some modifications [27,28]. The AIS (19 g) were sequentially extracted by hot water (20:1 (*w*/*v*)) (2 h at 80 °C), next by aqueous solution of ammonium oxalate 0.05 M (20:1 (*w*/*v*)) (4 h at 25°C, pH = 5), and finally, by 1 M NaOH aqueous solution (2 h at 4 °C, pH = 13). The alkaline extract was conducted in the presence of sodium borohydride NaBH4. The different extracts were filtered through cloth, precipitated by ethanol overnight at 4 °C to a volume ratio of 75% (v/v), and then centrifuged (3500 rpm, 15 min). The resulting precipitates were dissolved in distilled water, and they were deproteinized three times, following Savag’s method [29], to then be dialyzed extensively (14 kDa cut-off) against distilled water at 4 °C. Finally, they were lyophilized, resulting in water-soluble polysaccharide (WPL), chelator-soluble polysaccharide (CPL), and alkaline-soluble polysaccharide (BPL) (Figure 1). The yields of different extracts were calculated as follows:

Yield (%) = (m_0_/m) × 100 (1)

where m_0_ (g) and m (g) were the weights of lyophilized polysaccharides and leaf powder of *Allium roseum*, respectively.

### 2.4. Chemical Composition Analysis

The carbohydrate content of all the extracts was determined via the phenol-sulfuric acid method, using galactose as standard [30]. Galacturonic acid content was measured by a carbazole assay, using galacturonic acid as standard [31], and the protein content was appraised by the Lowry method [32].

### 2.5. Monosaccharide Composition Analysis

The monosaccharide composition of extracted polysaccharides was analyzed using Bartolozzi method [33]. Briefly, polysaccharides were hydrolyzed by TFA (2 M, 1 mL) at 70 °C for 2 h under a nitrogen atmosphere, and excess TFA was removed by distilled water. The aqueous solution of D-myo-inositol (100 μL, 1800 μg/mL) (internal standard) was added to the mixture. The hydrolyzed product was converted to its trimethylsilyl (TMS) derivative by mixing the dried sample with dry pyridine and N,O-bis (trimethylsilyl) trifluoroacetamide. The residue was analyzed by gas chromatography-mass spectrometry (Varian CP 3800) gas chromatograph (Varian, Inc. Santa Clara, CA, USA) equipped with DB-5 capillary column (30 m, 0.25 mm, and 0.25 μm film thickness).

### 2.6. UV, FTIR and NMR Analysis

The ultraviolet spectrum of each polysaccharide (1 mg/mL) was registered by Unico UV2100 spectrophotometer (USA), from 200 to 800 nm. The infrared (IR) spectra of different polysaccharides were obtained using a Fourier transform infrared spectrophotometer (Agilent Technologies/Gladi-ATR, Santa Clara, CA, USA), with a scan range from 400 to 4000 cm^−1^. Results were analyzed via the OriginPro 8 software. Prior to NMR-spectroscopic analysis, 1D and 2D NMR experiments were realized at 400 MHz with a Bruker Avance DPX-400 spectrometer. For analysis, lyophilized polysaccharides were dissolved in deuterium oxide (15 mg/mL), and the tetramethylsilane (TMS) was used as an internal standard.

### 2.7. Macromolecular Characterization

The macromolecular characteristics were determined using size exclusion chromatography (SEC) with three detectors, i.e., the multi-angle light scattering (MALS) (HELEOS II, Wyatt Technology, CA, USA), viscometer (VD) (Viscostar II, Wyatt Technology, CA, USA), and differential refractive index (DRI) (RID 10 A Shimadzu, Japan) detectors. The polysaccharides were solubilized in the eluent LiNO3 (0.1 mol/L) at 2 g/L then, filtered through membrane 0.45 μm, and injected (100 μL) with an automatic injector (SIL-20A, Shimadzu, Japan). The separation was made with two columns OHPAC 804HQ and 806HQ columns (Shodex, Japan) in series. The analysis was performed via a data processing Zimm [34] “order 1”, using angles from 34.8° to 142.8°. The corresponding value of dn/dc was 0.15 mL/g [35].

### 2.8. In Vitro Assay of Antioxidant Activity

#### 2.8.1. DPPH Free Radical Scavenging Activity

The DPPH free radical scavenging activity of different extracted polysaccharides was determined by the method of Hafsa et al. [36]. In summary, 500 µL of each extract at varying concentrations (5; 2.5; 1.25; 0.625 mg/mL) was mixed with 500 µL of DPPH ethanol solution (0.02%); then, was incubated for 30 min protected from light. The absorbance was filed at 515 nm, and the percentage of DPPH free radical scavenging was determined by Equation (2):

DPPH radical scavenging activity (%) = [1 − (A_1_ − A_2_)/A_0_] × 100 (2)

Herein, A_1_, A_2_, and A_0_ were the sample’s absorbance, sample’s absorbance without DPPH, and absorbance of the control (deionized water, instead of the sample), respectively.

#### 2.8.2. ABTS Radical Scavenging Activity

The ABTS radical scavenging activity of WPL, CPL, and BPL was assessed according to the specified method [37]. The ABTS radical was chemically generated by mixing a 4.95 mM potassium persulfate (K_2_S_2_O_8_) solution and ABTS (7 mM) solution and then stored in the dark for 12 h. The ABTS solution was diluted with ethanol to approximately 0.7 ± 0.02 absorbance at 734 nm wavelength. After preparing different concentrations for each sample, with distilled water (0.187, 0.375, 0.75, 1.5, and 3 mg/mL), 200 µL of ABTS solution was mixed with 20 µL of each concentration. The mixed solution was protected from light for 30 min and then measured the absorbance at 734 nm. ABTS radical scavenging activity was determined by Equation (3):

ABTS antioxidant activity (%) = [1 − (A_1_ − A_2_)/A_0_] × 100 (3)

Thus, A_1_, A_2_, and A_0_ were the sample’s absorbance, sample’s absorbance without ABTS, and absorbance of the control, respectively.

#### 2.8.3. Ferric Reducing Activity (FRAP)

The ferric reducing power of all polysaccharides was investigated using the reported method [38]. The different concentrations of each extract (0.187, 0.375, 0.75, 1.5, and 3 mg/mL) were combined with 500 µL of a phosphate buffer solution (0.2 M, pH 6.6) and 500 µL of a 1% potassium ferricyanide solution. The mixture was then incubated at 50 °C for 20 min before adding 500 µL trichloroacetic acid (10% w/v). After centrifugation (10 min), 500 µL of ferric chloride (0.1%) and 500 µL of distilled water were added to the supernatant solution and left at room temperature for 10 min. Then, the absorbance at 700 nm was measured using Vitamin C as positive standard. Ferric reducing activity was determined according to the following equation:

Reducing power = A_1_ − A_0_ (4)

Here, A_1_ and A_0_ were sample’s absorbance and absorbance without ferric chloride, respectively.

### 2.9. Hepatotoxicity Assay

#### 2.9.1. Animals

Adult male Swiss albino mice, weighing 20–25 g and aged 6-weeks-old, were obtained from central pharmacy of Tunisia and housed in cages at room temperature (22 ± 2 °C), with relative humidity of 55 ± 20%. The natural 12-h day/night cycle was respected. Food (pellet diet) and water ad libitum were available.

The animals were housed according to the Ethics and Confidentiality Committee (EEC) 609/86 directives regulating the welfare of experimental animals, and the experiments were approved by the local ethics committee of Institute of Biotechnology, University of Monastir (Ref: CER-VS/ISBM 022/2020).

#### 2.9.2. Cadmium Exposure and Sampling

After 7 days of acclimatization, animals were randomly divided into four groups (three mice for each): Group 1 used as control group, received 0.5 mL isotonic saline daily at 37 °C. Group 2 served as extract model group, each animal received 250 mg/kg b.w of WPL, CPL, or BPL daily. Group 3 served as toxin model, with a dose of 1 mg/kg b.w of CdCl_2_ in the last day. Finally, Group 4 received sample 250 mg/kg b.w daily and CdCl_2_ (1 mg/kg b.w) in the last day. In this work, we used a low dose of cadmium, compared to that usually used in the literature (4 mg/kg) [39]. In addition, Cd sulfate was conducted orally to mice by gavage because it is the main mode of exposure to Cd in humans and animals. All groups were performed once daily for 7 consecutive days. After 24 h fasting, following the last administration, mice were sacrificed, and livers were dissected and frozen in liquid nitrogen before storing at −80 °C for analysis. After centrifugation (15 min), the supernatants were stored at 4 °C for the biochemical analysis. The choice of the different samples doses was made based on works of Athmouni et al. [18].

### 2.10. Biochemical Analysis

#### 2.10.1. Total Protein Content

The liver tissue was homogenized by automatic homogenizer and then centrifuged at 1600× *g* for 15 min. The Bradford solution was added, mixed, and incubated with the supernatant during 15 min. The total protein concentration of liver homogenate (μg/mL) was measured at 595 nm using the method of Bradford, utilizing bovine albumin as the standard [40].

#### 2.10.2. Superoxide Dismutase Activity

Superoxide dismutase (SOD) levels were measured using the Marklund method [41], at an absorbance of 325 nm, to determine the inhibition of auto-oxidation of pyrogallol, and it was expressed as U/mg protein.

#### 2.10.3. Catalase Activity

Catalase (CAT) levels were essayed at 240 nm to determine the rate of decrease in H_2_O_2_ absorbance using the method of Clairbone [42]. One unit of CAT specific activity was determined as the amount of enzyme that catalyzes the degradation of 1 μmol H_2_O_2_/min/mg protein and is, therefore, expressed as μmol/min/mg protein.

#### 2.10.4. Lipid Peroxidation Measurement

Malondialdehyde (MDA) levels were determined by the 2-thiobarbituric acid (TBA) method [43]. It is expressed as μmol MDA/mg protein.

### 2.11. Data Analysis

Results were expressed as mean ± SEM (standard error of the mean). Treated animals were compared with the group control (untreated) using one-way ANOVA. Statistical differences were completed by student’s test using the Excel software program (16.0). Differences were considered significant at *p* ≤ 0.05.

## 3. Results

### 3.1. Isolation, Purification and Chemical Analyses of Polysaccharides

Three polysaccharides (WPB, CPB, and BPB) were sequentially extracted from the leaves of *Allium roseum*, as displayed in Figure 1. They differed in their yields, neutral sugar, and uronic acid content, as shown in Table 1. The water polysaccharides WPL (4.03%) presented the highest yields, compared to those of CPL (2.80%) and BPL (3.02). The obtained yields are in the same order of magnitude as those reported by Kratchanova [28] for leek extracts.

**Figure 2:**
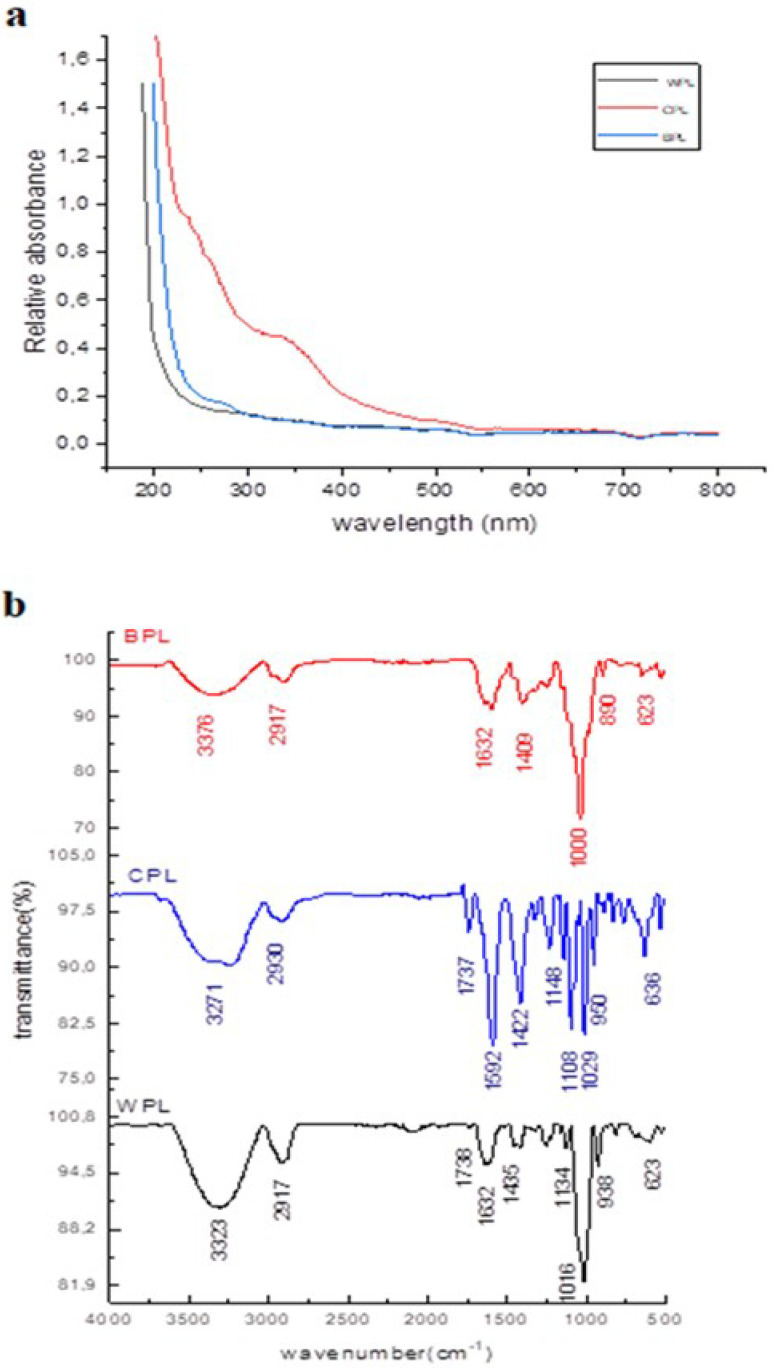
(**a**) UV-vis absorption and (**b**) FTIR spectra of WPL, CPL, and BPL extracted from *Allium roseum* leaves.

The UV spectrum (Figure 2a) showed the absence of absorption peak at 200–280 nm, demonstrating no protein or nucleic acid in all the extracts. The phenol-H_2_SO_4_ calorimetric assay showed that the content of total sugars was the most abundant element in all polysaccharides. Besides, they have significant uronic acid content: 34.64% (WPL), 29.27% (CPL), and 18.62% (BPL). The monosaccharide composition of all samples was determined by GC-MS. Results showed that WPL, CPL, and BPL were mainly composed of glucose (41.78%, 41.94%, and 50.37%), mannose (32.87%, 30.80%, and 27.03%), galactose (6.91%, 7.67%, and 3.98%), rhamnose (13.12%, 10.78%, and 13.31%), and xylose (3.31%, 4.61%, and 4.28%) respectively. FT-IR spectroscopy was used to determine the function present in the structure of WPL, CPL, and BPL. It is shown in Figure 2b. two bands characteristic of polysaccharides: a large absorption band between 3376–3200 cm^−1^ for O–H stretching vibrations and small absorption peak between 2990–3890 cm^−1^ for C–H stretching vibrations. An intense peak between 1632 and 1590 cm^−1^ was attributed to the band of carboxylate ion stretching (COO^-^) [44]. The weak absorption at around 1737 cm^−1^ was allotted to the C=O stretching vibration of the O-acetyl groups or esterified carboxyl group [45]. This finding suggests that WPL, CPL, and BPL were acidic polysaccharides. Furthermore, the peaks between 1100 and 1200 cm^−1^ were attributed to C-O-C glycosidic bonds, confirming the pyranose form of the different extracts [45]. Absorbance between 1000 and 1029 cm^−1^ was allotted to the vibration of C–O–H deformation. Absorbance at 950, 938, and 890 cm ^−1^ indicate the presence of β-glycosidic bonds in the polysaccharide, and the weak absorption bands at 819 cm^−1^ showed the presence of α-configuration [46]. These findings revealed that WPL, CPL, and BPL possess a typical absorption peak of polysaccharides, with both α- and β-configuration in the main chain.

**Figure 3:**
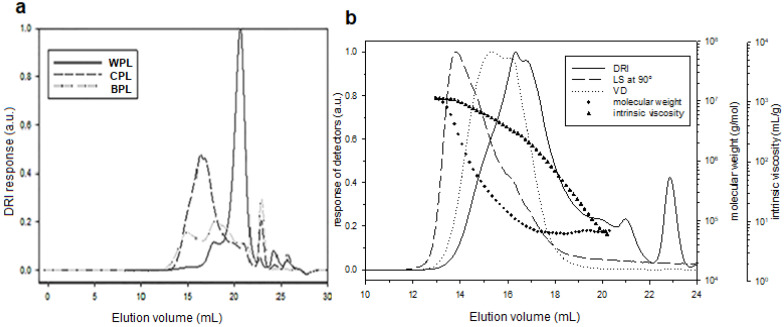
HPSEC analysis: (**a**) DRI elution profiles of WPL, CPL, and BPL in LiNO_3_ 0.1 mol/L. (**b**) Elution profiles obtained by SEC with refractive index, viscosity, and LS at 90° of CPL, together with molecular weights and intrinsic viscosity in LiNO_3_ 0.1 mol/L.

The three samples were analyzed by SEC with three detectors MALS, VD, and DRI online. To begin with, we have plotted (in Figure 3a) only the refractometric response, i.e., the concentration during elution. For WPL, the start of elution is later. With MALS and VD, it is possible to obtain the molecular weight and the intrinsic viscosity during the elution and calculate the average characteristics (Table 2).

As illustrated, we plotted the data for CPL (Figure 3b). WPL does have the lowest Mn value but with a very high polydispersity, due to the presence of some long chains. CPL and BPL have higher number average molecular weight, with a shorter polydispersity index.

### 3.2. NMR Characterization

Signals of each extract in 1D and 2D NMR spectra were assigned based on monosaccharide analysis and the chemical shifts noted in literature. The NMR data for extracted polysaccharides are noted in Table 3. As shown in Figure S1B, the presence of characteristic signal of uronic acid in the 13C NMR spectra (163 ppm) and absence of any signals in the region of δ C 82–88 confirmed that all the extracts are acidic polysaccharides with pyranose form. Usually, the chemical shifts of anomeric protons and carbons provide an idea regarding the α- and β-configuration. Indeed, the anomeric proton signals (Figure S1A) at 4.90–5.80 ppm were assigned to α configuration, and the 1H signals at 4.40–4.9 ppm referred to β-configuration [47]. 1H NMR spectra revealed four anomeric proton signals for each polysaccharide, at δ 4.50, 4.47, 5.21, and 5.18 ppm for WPL, δ 4.40, 4.44, 4.97, and 5.01 ppm for CPL, and δ 4.55, 4.44, 5.20, and 5.16 ppm for BPL, corresponded respectively to the H-1 of β-Manp, β-Glcp, α-GalpA, and α-Rhap. Furthermore, proton signals that appeared at 2.6 ppm are assigned to CH_3_ proton of O-Ac groups [48]. The resonances at δ 1.24 ppm corresponded to the methyl group of Rhap [49]. All the spectra showed five major signals, attributed to α-D-galacturonic acid residues: H-1 (5.21, 4.97, and 5.20 ppm), H-2 (3.77, 3.73, and 3.72) ppm, H-3 (4.01, 4.05, and 4.03 ppm), H-4 (4.21, 4.30, and 4.39 ppm), and H-5 (4.54, 4.80, and 4.89 ppm), respectively, for WPL, CPL, and BPL [50]. The 13C NMR spectra showed (Figure S1B) signals in the anomeric region, at δ 96.75, 98.17, 99.17, and 92.56 ppm for WPL, δ 98.64, 103.32, 101.84, and 93.52 ppm for CPL, and δ 96.61, 101.65, 99.34, and 92.57 ppm for BPL, characteristic of C-1 β-Manp, β-Glucp, α-GalpA, and α-Rhap, respectively [51,52]. The anomeric signals confirmed the presence of pyranose-form sugars commonly occurring in the region of 92–106 ppm. Moreover, signals in the range of 162 -165 ppm were referred to as the C-6 carboxyl group of uronic acid units. This last signal was allocated to non-methyl-esterified galacturonic acid residues. The absence of a signal of the methyl group of esterified galacturonic acid at δ 54.3 ppm confirmed this result. The existence of two carboxyl signals (162–163) may be due to the presence of branched and unbranched rhamnose residues in the backbone. In the HSQC spectra (Figure S2), it was possible to identify four cross peaks ^1^H/^13^C in the anomeric region of WPL for all residues: β-D-Manp (4.50/96.75), β-D-Glcp (4.47/98.17), α-D-GalpA (5.21/99.17), and α-L-Rhap (5.18/92.56) ppm. Similar to CPL, there were four signals ^1^H/^13^C in the anomeric region, centered at δ 4.40/98.64, 4.44/103.32, 4.97/101.84, and 5.01/93.52 ppm, which were assigned to β-D-Manp, β-D-Glcp, α-D-GalpA, and α-L-Rhap, respectively. The HSQC spectrum of BPL showed signals for four residues: β-D-Manp (4.55/96.61), β-D-Glcp (4.44/101.65), α-D-GalpA (5.20/99.34), and α-L-Rhap (5.16/92.57) ppm. The cross peaks of δ1.20/16.3 were easily identified as H-6/C-6 of the methyl group of α-L-Rhap. Additionally, the ^1^H/^13^C cross peaks centered at about 2.04/20.42 ppm proved the presence of O-acetyl groups in WPL, CPL, and BPL. The chemical shifts of H2/C2 to H6/C6, centered at 4.08/69.67, 3.75/72.44, 3.85/74.45, 3.50/74.45, and 3.88/63.35 ppm for WPL, were allocated to β-D-Manp [53]. Moreover, the groups H2-H6/C2-C6 around at 3.41–3.66/72.45–61.63 ppm were attributed to β-D-Glcp [50]. The ^1^H/^13^C HSQC spectra of CPL and BPL showed chemical shifts of glucose and mannose residues (Table 3), which are consistent with WPL data. COSY spectra (Figure S3) showed the cross-peaks at ~3.6/1.20 ppm of H-5/H-6 of Rhap. H1/H2 signals in WPL, CPL, and BPL, confirmed the existence of glucose, mannose, galacturonic acid, and rhamnose residue.

### 3.3. Antioxidant Activities

#### 3.3.1. DPPH Radical Scavenging Activity

**Figure 4:**
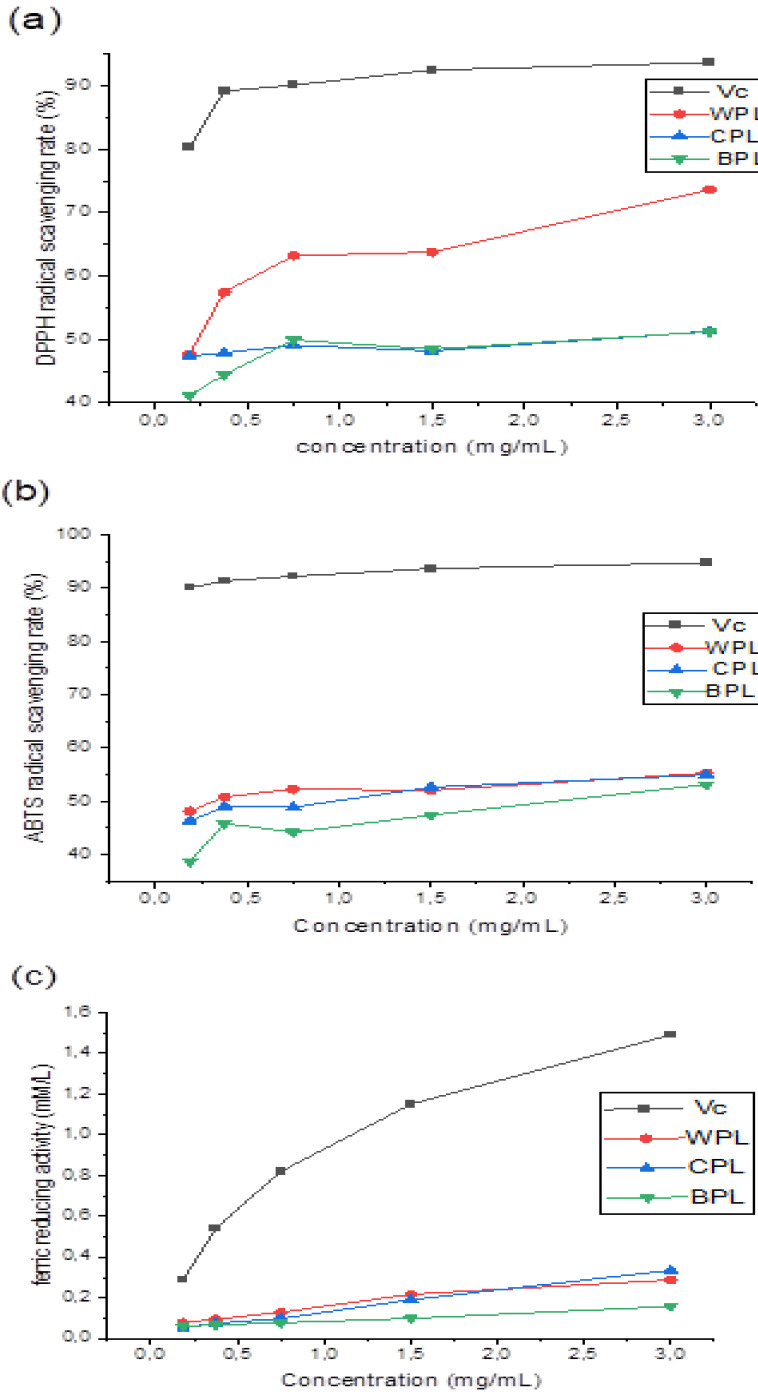
In vitro antioxidant activity of WPL, CPL, and BPL; (**a**) DPPH radical scavenging activity; (**b**) ABTS radical scavenging activity; (**c**) Ferric reducing activity.

Due to the relatively short time required for the analysis, the DPPH free radicals have been widely used to predict the antioxidant activity of several natural compounds. In the DPPH assay, the DPPH radical solution was reduced by adding the extract to the product diphenyl-picryl-hydrazine, and the color of the reaction mixture changed from purple to yellow. Our results showed that all extracts had a noticeable DPPH free radical scavenging activity, which was dependent on sample concentrations. At high concentration (3 mg/mL), the DPPH radical scavenging activities of WPL, CPL, and BPL were 73.66, 51.30, and 51.23%, respectively. It could be seen that WPL had the highest activity (Figure 4a). In comparison to previous reports, the extracts had better antioxidant activities than that of Zhang et al. [54], but they were still lower than the positive control (Vc).

#### 3.3.2. ABTS Radical Scavenging Activity

In the ABTS radical scavenging model, dark blue radical cations (ABTS^•+^) were reduced by antioxidants to colorless ABTS, which could be measured spectrophotometrically. As shown in Figure 4b, the antioxidant activities of WPL, CPL, and BPL increased with the increase of sample concentration up to 55.33%, 54.97%, and 53.15%, at a concentration of 3 mg/mL, respectively. This finding demonstrated that the percentage of inhibition of different extracts on ABTS radicals was better than that of Amamou et al. from Opuntia macrorhiza fruit peels [55]. Results showed that the extracted polysaccharides have satisfactory ABTS radical scavenging activity.

#### 3.3.3. Ferric Reducing Power

FRAP antioxidant activity assay is an important method that is widely used to test the antioxidant activities of drugs, based on reducing a ferric salt Fe^3+^ to ferrous salt Fe^2+^ by an antioxidant via an electron transfer reaction [56]. This reaction produces a blue color, whose intensity can be measured spectrophotometrically. In this study, the reducing activities of extracted polysaccharides were determined. The results showed a dose-dependent relationship between the reducing power and sample concentration. Here, the antioxidant activities of WPL, CPL, and BPL increased with increasing concentration (Figure 4c). It could be seen that WPL and CPL had a similar activity, with an approximate value of 0.33 and 0.29 mM/L, which were much lower than ascorbic acid.

### 3.4. Capacity of Hepatoprotective Activities In Vivo

**Figure 5:**
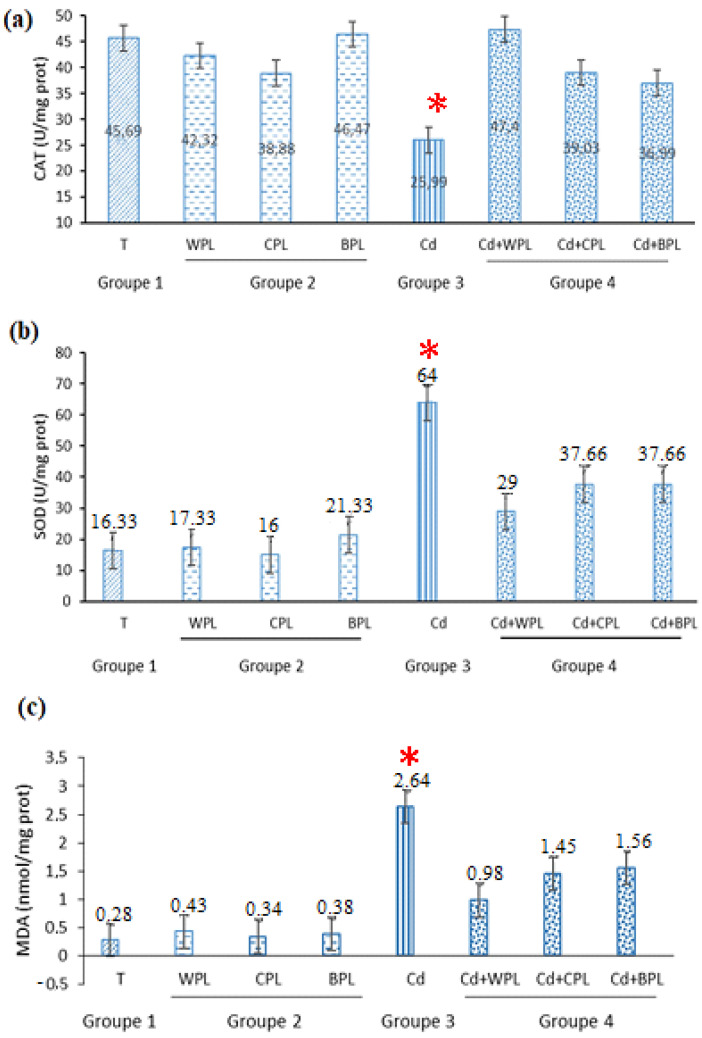
Effect of different polysaccharides on (**a**) CAT activity, (**b**) SOD activity, and (**c**) MDA level in liver tissue. Values are presented as mean ± SD of four groups. * Values are significantly different from the control at *p* ≤ 0.05.

In order to appreciate the potential antioxidant impact activity of different polysaccharides in vivo, the antioxidant biomarkers (SOD and CAT) and indicator of lipid peroxidation (MDA) were examined. Results showed (Figure 5a) a notable decrease of CAT activity when animals were exposed to the cadmium. Our investigation revealed that the highest level of SOD activity was obtained with Cd-treated animals (Figure 5b). As shown in Figure 5, in mice pretreated with different polysaccharides, the effects of Cd in levels of SOD, CAT, and MDA were attenuated. Polysaccharides pretreatment increased CAT and decreased MDA and SOD, as compared with those values of group 3 (toxin model). Nevertheless, treatment with polysaccharides alone did not show any difference in all biomarkers, when compared to the group 1 (control).

## 4. Discussion

*Allium* has a long history in both medicinal and food use in Tunisia. Many research demonstrated that *Allium* genus has various medicinal properties, due to its bioactive compounds (polyphenols and polysaccharides) [57]. Polysaccharides have received increasing attention, due to their various biological activities, such antioxidants, antidiabetic, and anti-tumor activities. Recently, research has been conducted to study the renoprotective and hepatoprotective effects of natural polysaccharides [58,59]. In the present paper, we showed that the polysaccharides extracted from *Allium roseum* have a protective effect against Cd-intoxication, which could be related to the free radical scavenging ability of the active molecules present in these polysaccharides. Three polysaccharides, i.e., WPL, CPL, and BPL, were obtained by sequential extraction in aqueous, chelating, and basic medium. Chemical analysis showed that these polysaccharides might be classified as acidic polysaccharides. Hence, they probably have antioxidants properties, according to previous reports [60]. Monosaccharides analysis suggested that the backbone of all extracts is mainly composed of glucose, mannose, and galacturonic acid. Rhamnose, xylose, and galactose may be in the position of branched structure. This is in agreement with previous reports, which demonstrated that onion polysaccharides consisted of glucose, mannose, galactose, xylose, and rhamnose [61]. Besides, Liu et al. isolated an acidic heteropolysaccharide composed mainly of glucose and galacturonic acid [62]. On the other hand, the monosaccharides composition of WPL, CPL, and BPL were different from the polysaccharide compounds of the welsh onion, i.e., fructose and glucose [63]. The highest amount of glucose, particularly in basic polysaccharide (BPL), can be attributed to the reduction of the uronic acid part into glucose by the NaBH_4_ [50]. Furthermore, macromolecular characterization revealed that the first hot water extraction solubilizes mostly the shorter chains. The next two extractions, in the presence of the chelating and alkaline agents, allow for the solubilization of the longest chains by breaking interactions for CPL and des-esterification for BPL. Hence, these media solubilized chains affect the viscosity more significantly. The presence of NaBH_4_ during the alkaline extraction has a preventive effect on the chain degradation. The in vitro and in vivo antioxidant activities of polysaccharides are heavily dependent on their structure and conformation. Our physico-chemical characterization proved that the extracted polysaccharides, i.e., WPL, CPL, and BPL, are acidic heteropolysaccharides with similar structural characteristics, which are composed of linked blocks of β-D-Glcp, β-D-Manp, and α-D-GalpA substituted with linear and branched rhamnose, galactose, and xylose residues.

The antioxidant capacities of different polysaccharides in vitro were tested, and the results showed that WPL, CPL, and BPL exhibited a significant dose-dependent antioxidant capacity, which could be allotted to the presence of numerous hydroxyl groups and uronic acid in polysaccharides structure [64]. The enzymatic antioxidants SOD and CAT are considered important defense systems against oxidative stress [18], since they are the first line of defense against oxygen reactive species. They could transform superoxide to peroxide and then to H_2_O and O_2_. In addition, they could stop the chain reaction of lipid peroxidation [65]. According to Wang et al., decreased CAT activity is probably due to the inactivation or change in the assembly of enzyme subunits [66]. On the other hand, the low CAT activity could be due to the high rate of Cd accumulation in the hepatic tissue and possible failure in removing of H_2_O_2_. Furthermore, the results showed that the SOD levels decreased with Cd exposure. This result is closely associated with the hypothesis that metal enhances free radical production, which stimulates SOD activity [5]. This could be considered an adaptive response to protect cell against oxidative stress. CAT activity is habitually correlated to SOD activity, and both enzymes function together and constitute the first line of defense against oxidative stress. The main end of the present study is the key role played by the different PS extracts in modulating the two enzymes’ activities. One may assume that polysaccharides exerted an immediate protective effect against the pro-oxidant impact of the metal, in view of antioxidant biomarker rates. Belhaj et al. reported that Cd stress significantly increased the MDA levels in liver homogenate of mouse treated with Cd [59], which is in agreement with our results. Altogether, the results, relative to antioxidant enzyme activities and MDA production of group 4 (animals treated by cadmium combined with polysaccharides), confirm our hypothesis that the different extract has an hepatoprotective effect against Cd intoxication. This could be correlated with the radical scavenging and antioxidant activities of active groups present in the different extracts, such as OH groups. Moreover, the present study proposes that WPL treatment happens to offer the best protection against Cd-induced liver damage, compared to CPL and BPL. This finding suggests that the molecular weight largely affects the antioxidant properties, which was proved by previous researches [67]. CPL and BPL must be slightly degraded to be used for this application [68]. Herein, the findings suggest that polysaccharides extracted from *Allium roseum* leaves could be developed as a potential natural antioxidant.

## 5. Conclusions

In this work, new acidic heteropolysaccharides were extracted sequentially in three media from *Allium roseum* leaves. The structural characterizations revealed that the different polysaccharides were mainly composed of linked blocks of β-D-Glcp, β-D-Manp, and galacturonic acid substituted with rhamnose, galactose, and xylose. Moreover, these polysaccharides showed noticeable in vitro antioxidants and strong in vivo hepatoprotective activities against Cd injection. The polysaccharides extracted in water medium displayed the highest activities, which is due to its low mass molecular weight. The findings suggested that the polysaccharides extracted from leaves of *Allium roseum* could be used as a natural antioxidant to enhance human health.

## Figures and Tables

**Table 1:** Chemicals analyses and monosaccharide composition of extracted dry samples ^a^.

Samples	WPL	CPL	BPL
Yield (%)	4.03	2.80	3.02
Neutral sugar (%)	65.40 ± 0.22	70.94 ± 0.29	81.35 ± 0.32
Uronic acid (%)	34.64 ± 0.35	29.27 ± 0.20	18.62 ± 0.24
Protein (%)	n.d ^b^	n.d ^b^	n.d ^b^
Neutral monosaccharide composition (mol %)			
Rhamnose	13.12	10.78	13.31
Galactose	6.91	7.67	3.98
Glucose	41.78	41.94	50.37
Mannose	32.87	30.80	27.03
xylose	3.31	4.61	4.28

^a^ WPL, CPL, and BPL: polysaccharides extracted from leaves of *Allium roseum* under water, chelating, and basic medium. ^b^ n.d: not detected.

**Table 2:** Macromolecular characteristics of extracted samples.

Samples	WPL	CPL	BPL
M_w_ (g/mol)	600,000 ± 5000	240,000 ± 10,000	660,000 ± 20,000
M_n_ (g/mol)	40,000 ± 3000	95,000 ± 6000	160,000 ± 10,000
[ղ] (mL/g)	11.5 ± 1	257 ± 9	62.1 ± 0.6
ÐIp^b^ = Mw/Mn	15	2.5	4.1
R_H_ (nm)	5.6 ± 0.3	18± 1	26 ± 2

**Table 3:** Chemical shifts for the resonances of glycosyl residues of water WPL, chelating CPL, and basic BPL polysaccharides in ^1^H and ^13^C NMR spectra.

WPL:							
Glycosyl Residues		Chemical Shifts, δ (ppm)					
		H1/C1	H2/C2	H3/C3	H4/C4	H5/C5	H6/C6
M	-β-D-Manp	4.50/96.75	4.08/69.67	3.75/72.44	3.85/74.45	3.50/74.45	3.88/63.35
G	-β-D-Glcp	4.47/98.17	3.41/72.45	3.54/74.45	3.69/80.67	3.45/72.45	3.66/61.67
A	-α-D-GalpA	5.21/99.17	3.77/69.18	4.01/69.67	4.21/80.67	4.54/72.45	- 163.69–163.38
R	-α-L-Rhap	5.18/92.56	4.01/74.45	3.85/69.1	3.48/71.85	3.62/68.46	1.24/16.30
CPL:							
Glycosyl Residues		Chemical Shifts, δ (ppm)					
		H1/C1	H2/C2	H3/C3	H4/C4	H5/C5	H6/C6
M	-β-D-Manp	4.40/98.64	4.05/69.29	3.73/72.52	3.48/68.82	3.48/76.80	3.67/61.02
G	-β-D-Glcp	4.44/103.32	3.44/71.40	3.58/74.61	3.49/80.22	3.49/74.61	3.85/62.93
A	-α-D-GalpA	4.97/101.84	3.73/68.82	4.05/69.81	4.30/80.22	4.80/72.52	- 162.81–163.17
R	-α-L-Rhap	5.01/93.52	4.30/76.80	3.58/71.4	3.85/69.81	3.56/69.29	1.20/16.73
BPL:							
Glycosyl Residues		Chemical Shifts, δ (ppm)					
		H1/C1	H2/C2	H3/C3	H4/C4	H5/C5	H6/C6
M	-β-D-Manp	4.55/96.61	4.10/71.49	3.71/72.75	3.66/76.32	3.45/76.78	3.69/62.95
G	-β-D-Glcp	4.44/101.65	3.47/73.64	3.64/75.81	3.66/76.78	3.42/76.32	3.64/62.95
A	-α-D-GalpA	5.20/99.34	3.72/69.17	4.03/69.21	4.39/nd	4.89/71.80	- 163.20–162.8
R	-α-L-Rhap	5.16/92.57	4.14/76.78	3.64/70.92	3.55/69.21	3.40/69.17	1.36/nd

## Data Availability

Data is contained within the article and supplementary material.

## Supplementary Materials

The following supporting information can be downloaded at: https://www.mdpi.com/article/10.3390/antiox11101866/s1, Figure S1 1H (A) and 13C (B) NMR spectrum of the polysaccharides extracts WPL, CPL and BPL. Figure S2 The 1H/13C HSQC spectrum: (a) overall spectrum WPL; (b) close-up spectrum WPL; (c) overall spectrum CPL; (d) close-up spectrum CPL; (e) overall spectrum BPL; (f) close-up spectrum BPL. Figure S3 1H-1H COSY: (a) overall spectrum WPL; (b) close-up spectrum WPL; (c) overall spectrum CPL; (d) close-up spectrum CPL; (e) overall spectrum BPL; (f) close-up spectrum BPL.
