# Supplementary material for: Characterization of Polysaccharides Sequentially Extracted from Allium roseum Leaves and Their Hepatoprotective Effects against Cadmium Induced Toxicity in Mouse Liver
Source: Antioxidants (Basel). 2022 Sep 21;11(10):1866. doi: 10.3390/antiox11101866 (PMC9599006; doi:10.3390/antiox11101866)
Supplement: Supplementary file 1 [file antioxidants-11-01866-s001.zip › antioxidants-1889586-supplementary.pdf]

Article

Characterization of Polysaccharides Sequentially Extracted from *Allium roseum* Leaves and their Hepatoprotective Effects against Cadmium Induced Toxicity in Mouse Liver

Nesrine Teka [1], Fahad M. Alminderej [2,*], Ghada Souid [3], Yassine El-Ghoul [2,4,*], Didier Le Cerf [5] and Hatem Majdoub [1,*]

[1] Laboratory of Interfaces and Advanced Materials, Faculty of Sciences of Monastir, University of Monastir, Monastir 5000, Tunisia
[2] Department of Chemistry, College of Science, Qassim University, Buraidah 51452, Saudi Arabia
[3] Research Unit: Mycotoxins, Phycotoxins and Associated Pathologies, Faculty of Pharmacy, University of Monastir, Monastir 5000, Tunisia
[4] Textile Engineering Laboratory, University of Monastir, Monastir 5019, Tunisia
[5] Normandie University, UNIROUEN, INSA Rouen, CNRS, PBS, UMR 6270 & FR 3038, 76000 Rouen, France
* Correspondence: f.alminderej@qu.edu.sa (F.M.A.); y.elghoul@qu.edu.sa (Y.E.-G.); hatemmajdoub.fsm@gmail.com (H.M.)

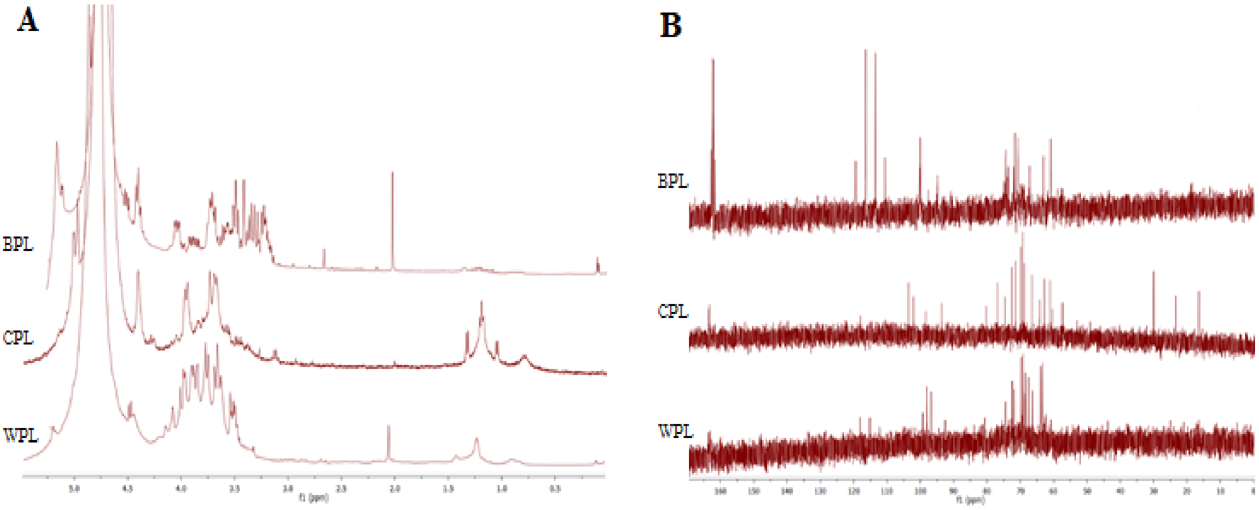

Figure S1 ^{1}H (A) and ^{13}C (B) NMR spectrum of the polysaccharides extracts WPL, CPL and BPL.

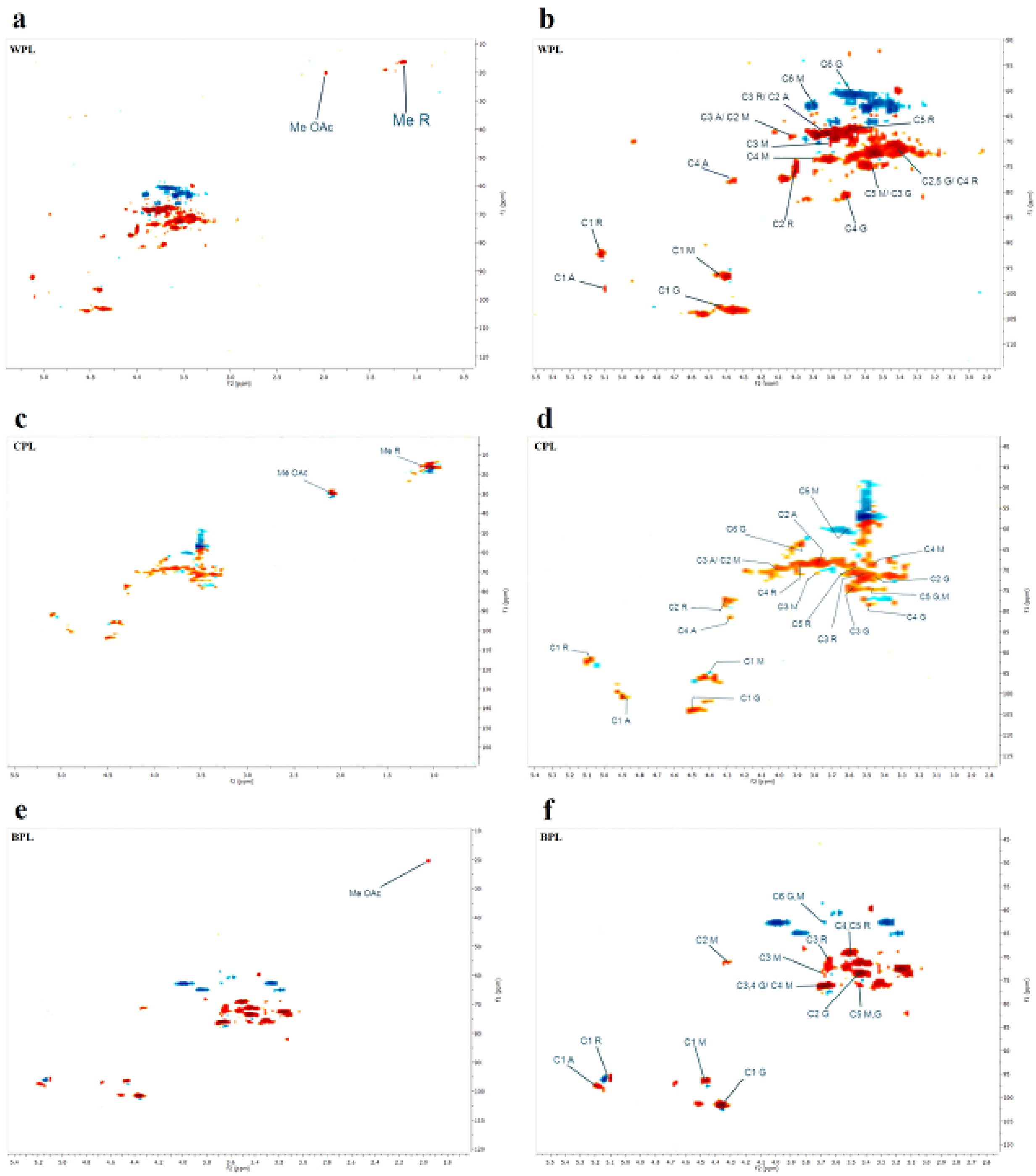

Figure S2 The $^{1}H/^{13}C$ HSQC spectrum: (a) overall spectrum WPL; (b) close-up spectrum WPL; (c) overall spectrum CPL; (d) close-up spectrum CPL; (e) overall spectrum BPL; (f) close-up spectrum BPL.

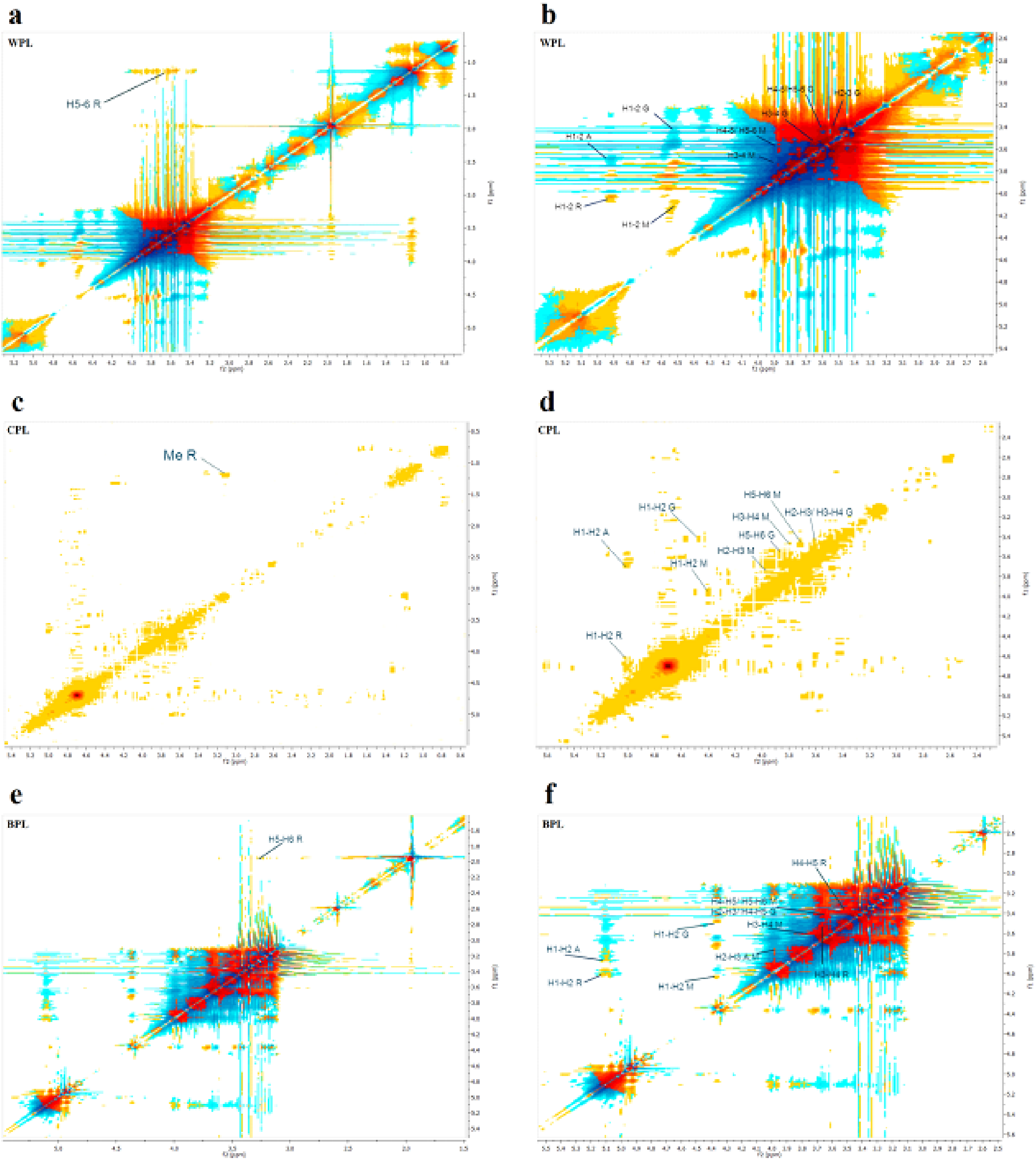

Figure S3 ^{1}H-^{1}H COSY: (a) overall spectrum WPL; (b) close-up spectrum WPL; (c) overall spectrum CPL; (d) close-up spectrum CPL; (e) overall spectrum BPL; (f) close-up spectrum BPL.
